# Genetic testing for *SCA27B* in Korean multiple system atrophy

**DOI:** 10.1093/brain/awaf263

**Published:** 2025-07-17

**Authors:** Joshua Laß, Michele Berselli, Doug Rioux, Susen Schaake, Jordan Follett, Jonathan E Bravo, Alexander D Veit, William Ronchetti, Sarah B Reiff, Matthew J Huentelman, Dana Vuzman, Pamela Bower, Vikram Khurana, Joanne Trinh, Beomseok Jeon, Han-Joon Kim, Matthew J Farrer

**Affiliations:** Institute of Neurogenetics, University of Lübeck, Lübeck 23538, Germany; Department of Biomedical Informatics, Harvard Medical School, Boston, MA 02115, USA; Department of Biomedical Informatics, Harvard Medical School, Boston, MA 02115, USA; Institute of Neurogenetics, University of Lübeck, Lübeck 23538, Germany; McKnight Brain Institute, Department of Neurology, College of Medicine, University of Florida, Gainesville, FL 32611, USA; McKnight Brain Institute, Department of Neurology, College of Medicine, University of Florida, Gainesville, FL 32611, USA; Department of Biomedical Informatics, Harvard Medical School, Boston, MA 02115, USA; Department of Biomedical Informatics, Harvard Medical School, Boston, MA 02115, USA; Department of Biomedical Informatics, Harvard Medical School, Boston, MA 02115, USA; Neurogenomics Division, Translational Genomics Research Institute, Phoenix, AZ 85004, USA; Department of Biomedical Informatics, Harvard Medical School, Boston, MA 02115, USA; Division of Genetics, Department of Medicine, Brigham and Women’s Hospital, Harvard Medical School, Boston, MA 02115, USA; Mission MSA (Formerly MSA Coalition), McLean, VA 22102, USA; Division of Movement Disorders, Department of Neurology, Brigham and Women’s Hospital, Harvard Medical School, Boston, MA 02115, USA; Institute of Neurogenetics, University of Lübeck, Lübeck 23538, Germany; Department of Neurology, Seoul National University Hospital, Seoul National University College of Medicine, Seoul 03080, Korea; Department of Neurology, Seoul National University Hospital, Seoul National University College of Medicine, Seoul 03080, Korea; McKnight Brain Institute, Department of Neurology, College of Medicine, University of Florida, Gainesville, FL 32611, USA

Recently, Chelban *et al*.^1^ reported that FGF14 GAA_≥250_ repeat expansions impact progression and survival in multiple system atrophy (MSA). They identified 2.89% (*n* = 19/657) in MSA versus 1.40% (*n* = 12/1003) in controls. These 19 cases were reported to have faster progression, and a repeat size that was inversely correlated with survival.

To extend and validate these results, we comprehensively examined all genetic variability in the *FGF14* locus in MSA in an ongoing longitudinal study from South Korea.^2,3^ Patients were enrolled in the Seoul National University Hospital (SNUH) outpatient clinic setting from 2012 to 2021. Patients with ‘probable MSA’, according to the second consensus criteria, who provided written informed consent, were eligible to participate. The study was approved by institutional review boards (IRB) at SNUH (H-1601-048-733) and the University of Florida (#IRB202000632) to enable whole genome sequencing (WGS). Our cohort included 199 individuals (91 male, 108 female) with a mean age at examination of 58.2 ± 8.3 (median 58, range 34–79), of which 122 patients had MSA-cerebellar type (MSA-C) and 77 MSA-Parkinson’s type (MSA-P) (Supplementary Table 1).^3^ The minimum mean sequencing depth for all samples was 35x (Supplementary material, ‘Methods’ section). All data have been shared with the MSA Coalition Collaborative Core Network (https://missionmsa.org/). Additional anonymized genomic data has also been made available from healthy Korean volunteers participating in the Korean Genome Project (KGPcontrols, *n* = 1048).^4^

All non-synonymous single nucleotide variants (SNVs) and small insertions and deletions (indels) in *FGF14* were investigated. None were consistent with SCA27A, and no variants have a known pathogenic or likely pathogenic annotation in Clinvar.^5^ All genomic variability observed in *FGF14* in MSA was also examined in jointly called WGS data from the KGPcontrols.^4^ One MSA-C patient has a rare coding variant observed in exon 5 c.707C>T (p.Ala326Val; NC_000013.11:g.101722883G>A; MAF KGPcontrols = 0.0019, gnomADEastAsia = 0.0002), whereas 10 patients had rare indel variants of uncertain significance (VUS) within the 3ʹ-untranslated region (Supplementary Table 2). We interrogated all MSA genomes for *FGF14* 5ʹ-flanking (GAA)*_n_* repeats with the ExpansionHunter algorithm.^6^ The (GAA)*_n_* triplet repeat number was 17.8 ± 16.2 SD in Korean MSA patients versus 17.9 ± 17.2 SD in KGPcontrols. The most frequent (GAA)*_n_* repeat number per allele was 10 (27.4%). Comparable results and minor allele frequencies (MAF) were predicted for all motifs in cases and controls (Supplementary Fig. 1). However, we sought to validate allele sizes based on ExpansionHunter results with PCR-based amplicon length analysis. *FGF14* (GAA)*_n_* locus PCR was subsequently performed in 199 MSA patients and 196 ethnically matched control participants (Fig. 1). The overall distributions in control individuals [mean = 37 ± standard deviation (SD) 57 bp] and MSA (mean = 44 ± SD 68 bp) were not significantly different (*P* > 0.07). The majority of allele sizes in both groups were <50 bp including 89.2% (350/392) of controls and 86% (296/344) of MSA. However, intermediate sized FGF14_>150_ alleles were found in 13.4% (46/344) of MSA (mean = 195 ± SD 80 bp) and 6.9% (27/392) of controls (mean = 224 ± SD 64 bp). The threshold for intermediate repeat expansion is >250, and alleles with less GAA repeats should be considered in the normal range. As amplicon PCR with gel sizing and ExpansionHunter results were inconsistent, we used linear regression to assess their relationship (*P* = 0.18, *R*^2^ = 0.07). A subset of expanded alleles (*n* = 11) was also interrogated using long-read sequencing. The median quality of reads (phred score) was q = 13.4, and the median read length (including both alleles) was 0.934 kb. The mean read coverage was 63 411x. The results of the repeat length were more consistent with amplicon PCR gel sizing results (*P* = 0.75 × 10^−4^, *R*^2^ = 0.82). It was possible to detect a complex repeat interruption pattern in 10/11 patients with the long expansion (GCAGAAGAAGAAGAA)*_n_*(GCAGAA)*_n_*(GAA)*_n_*(GAG) (Supplementary Table 2). These patients were comparable to the cohort overall and none had atypical clinical features (Supplementary Table 1). In contrast to the PCR, long-read sequencing did not correlate with ExpansionHunter for (GAA)*_n_* expansion size (*P* = 0.052, *R*^2^ = 0.29).

**Figure 1:**
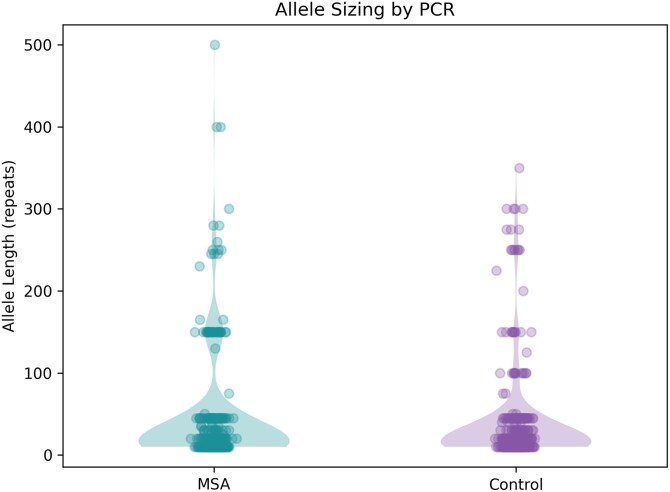
**FGF14 (GAA)*_n_* locus PCR in patients with multiple system atrophy and ethnically-matched control participants**. Allele length in triplet repeats as estimated from gel migration. MSA = multiple system atrophy.

In summary, *FGF14* haploinsufficiency and loss-of-function clearly contribute to spinocerebellar ataxia type 27A (SCA27A) and SCA27B.^7-9^ Nevertheless, in this Korean sample of patients with MSA there is no evidence for pathogenic genetic variability within the *FGF14* locus. Our results exclude *FGF14* (GAA)_>300_ pathogenic repeat expansions and rare *FGF14* coding variants. However, we do observe a higher frequency of intermediate FGF14 (GAA)_>150_ expanded alleles in patients (13.4%, 46/344) compared to controls (6.9%, 27/392). While intermediate size FGF14_<250_ repeats are not considered pathogenic, and this sample size is too small for significance, further reporting across a range of repeat sizes with meta-analysis may yet reveal some relationship with MSA susceptibility. Amplicon PCR gel sizing and long-read sequencing were highly correlated, but the former is not a precise tool for analysing exact repeat lengths nor repeat interruptions (Supplementary Table 2). Unfortunately, ExpansionHunter was not able to accurately annotate long, complex expanded repeats. Thus, we recommend amplicon PCR analysis for future genetic testing to highlight potential (GAA)_>250_ repeat expansions, with subsequent confirmation by long-read sequencing.

## Supplementary Material

awaf263_Supplementary_Data

## Data Availability

All genome data has been shared with the MSA Coalition Collaborative Core Network (https://missionmsa.org/). This is available on request through the corresponding author, and with appropriate data use agreements and Institutional Review Board approvals. Additional anonymized genomic data is available from healthy Korean volunteers participating in the Korean Genome Project (KGPcontrols, *n* = 1048) through the Korean National Institutes of Health.^4^
