# Supplementary material for: Genetic testing for SCA27B in Korean multiple system atrophy
Source: Brain. 2025 Jul 17;148(12):e109–11. doi: 10.1093/brain/awaf263 (PMC12677904; doi:10.1093/brain/awaf263)
Supplement: awaf263_Supplementary_Data [file awaf263_supplementary_data.pdf]

**Supplementary table 1. Clinical characteristics of patients with MSA and control series**

| Diagnosis | Count | Female | Male | Age, at diagnosis (years) | Cerebellar ataxia | Parkinsonism |
|---|---|---|---|---|---|---|
| MSA-C | 122 | 62 | 60 | 59.7 ± 9.4 | 122 | 42 |
| MSA-P | 77 | 46 | 31 | 57.4 ± 7.4 | 15 | 77 |
| *FGF14* expanded* | 10 | 5 | 5 | 57.8 ± 8.4 | 6 | 6 |
| Controls | 196 | 117 | 79 | 64.4 ± 9.0 | - | - |
| KGP controls[9] | 1,048 | - | - | - | - | - |

* MSA with expanded *FGF14* alleles from Table 2.

**Supplementary table 2. Repeat interruptions identified in expanded alleles via long-read sequencing**

| ID | long-read | Expanded allele length PCR | ExpansionHunter (total repeats) | Repeat interruption |
|---|---|---|---|---|
| **SNUH114** | 309 | 260 | 465 | $(GAA)_{31}(GCAGAAGAAGAAGAA)_{37}(GCAGAA)_{26}(GAA)_{20}(GAG)$ |
| **SNUH32** | 295 | 280 | 468 | $(GAA)_{21}(GCAGAAGAAGAAGAA)_{37}(GCAGAA)_{26}(GAA)_{22}(GAG)$ |
| **SNUH133** | 294 | 245 | 382 | $(GAA)_{26}(GCAGAAGAAGAAGAA)_{37}(GCAGAA)_{26}(GAA)_{22}(GAG)$ |
| **SNUH147** | 393 | 230 | 295 | $(GAA)_{27}(GCAGAAGAAGAAGAA)_{37}(GCAGAA)_{26}(GAA)_{22}(GAG)$ |
| **SNUH88** | 306 | 250 | 416 | - |
| **SNUH14** | 293 | 250 | 431 | $(GAA)_{21}(GCAGAAGAAGAAGAA)_{37}(GCAGAA)_{26}(GAA)_{22}(GAG)$ |
| **SNUH71** | 296 | 245 | 453 | $(GAA)_{21}(GCAGAAGAAGAAGAA)_{37}(GCAGAA)_{26}(GAA)_{22}(GAG)$ |
| **SNUH87** | 635 | 500 | 345 | $(GAA)_{107}(GCAGAAGAAGAAGAA)_{37}(GCAGAA)_{26}(GAA)_{22}(GAG)$ |
| **SNUH118** | 293 | 250 | 507 | $(GAA)_{21}(GCAGAAGAAGAAGAA)_{37}(GCAGAA)_{26}(GAA)_{22}(GAG)$ |
| **SNUH124** | 291 | 245 | 465 | $(GAA)_{20}(GCAGAAGAAGAAGAA)_{37}(GCAGAA)_{26}(GAA)_{20}(GAG)$ |
| **SNUH161** | 295 | 250 | 408 | $(GAA)_{21}(GCAGAAGAAGAAGAA)_{37}(GCAGAA)_{26}(GAA)_{22}(GAG)$ |

**Supplementary table 3.** ***FGF14*** **repeat motif catalogue examined with Expansion Hunter**

| **Position (hg38)** | **Gene** | **Motif** |
|---|---|---|
| chr13:102161575-102161724 | FGF14 | (GAA)* |
| chr13:102161575-102161724 | FGF14 | (CAG)* |
| chr13:102161575-102161724 | FGF14 | (GAAGAAGAAGAAGCA)* |
| chr13:102161575-102161724 | FGF14 | (GAAGAAGCA)* |
| chr13:102161575-102161724 | FGF14 | (GAAGAG)* |
| chr13:102161575-102161724 | FGF14 | (GAAGAGGAG)* |
| chr13:102161575-102161724 | FGF14 | (GAAGCA)* |
| chr13:102161575-102161724 | FGF14 | (GAAGGA)* |

**Supplementary figure 1. Analysis of separate** ***FGF14*** **repeat motifs and sizes, in MSA cases and controls, estimated with Expansion Hunter**

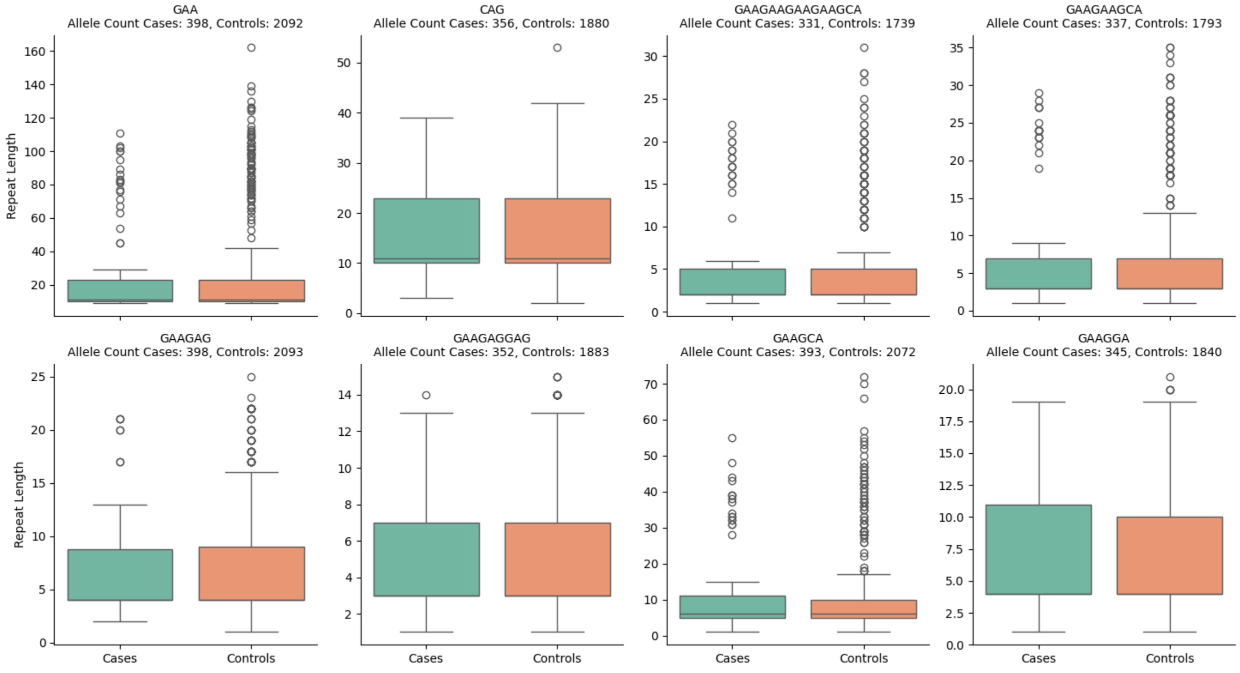

**Supplementary methods**

**Sample collection and whole genome sequencing (WGS)**

A detailed medical and family history was collected during the patient interview and recorded in the electronic medical record (EMR). Their EMR was also reviewed to obtain participants' full medical history. Following enrollment, 10ml peripheral blood was collected for biobanking. Genomic DNA was extracted from blood using QIAamp™ protocols and quantified by fluorescence on an Invitrogen Qubit Fluorometer. Subsequent WGS was performed by the Clinical & Translational Genomics Program at the University of Florida. Samples and data are processed and stored according to HIPAA compliance requirements, following CAP guidelines[1] and CLIA standards for quality and competence[2] at UF Health Medical Laboratories. Testing has been benchmarked using the National Institute of Standards and Technology 'Genome in a Bottle' Consortium standards (HG002 son, HG003 & HG004 parental genomes).[3]

Individually-indexed genomic libraries were prepared using dual unique indexes from ~200ng DNA/individual (New England Biolabs NEBNext® UltraTM II DNA Library Prep Kit for Illumina®). Genome library quality and quantity were confirmed by automated electrophoresis on an Agilent 2100 bioanalyzer and by qPCR. Individual libraries were then normalized and pooled in equimolar ratios for 2× 150 bp paired-end sequencing at 35X depth on an Illumina NovaSeq 6000. To enable code sharing and ensure security and PHI/HIPAA compliance, we maintain a containerized compute environment in Amazon Elastic Compute Cloud (AWS EC2) and a versioned bioinformatic pipeline for genome variant calling, including TrimmomaticPE, FastQC, and MultiQC. Initial quality control (QC) reports are generated and examined for all individual samples and batched runs. Data passing QC is transferred to CGAP via AWS protocols for secondary QC and bioinformatic analyses.

**Genomic PCR**

Published methods were used to amplify the *FGF14* (GAA) locus [16].[4] PCR primers 5'-AGCAATCGTCAGTCAGTGTAAGC (FGF14 Forward) and 5'-CAGTTCCTGCCCACATAGAGC (FGF14 Reverse) span the interval to give a 315bp product.

**Long-read sequencing (LRS)**

Expanded samples were selected to characterize the repeat domain of *FGF14*. Amplicon PCR products were visualized using the 4200 Tapestation and underwent purification using SPRI beads to isolate a targeted region of >500bp. The Native 24 Barcoding Kit (SQK-NBD114.24) was used for multiplexing the PCR products, and 1 μg of amplified PCR product was used as input. The following sequencing adapter ligation was performed with the SQK-NBD114.24 (ONT). The input for the library preparation was 200 fmol of DNA per sample. The final product was then loaded on R10 PromethION flow cells on a P24. Base-calling was performed with the high-accuracy model of Guppy version 7.1.4. The repeat length and interruptions were explored as previously described.[5,6] Only reads with an alignment length over 1kb were included in the analysis.

**Bioinformatic analysis**

The bioinformatics analysis was performed using CGAP pipelines and automatically executed in AWS cloud infrastructure using Tibanna, following Genome Analysis Toolkit (GATK) best practices.[7] Paired-end FASTQ files were aligned to GRCh38 reference genome using bwa-mem, followed by cleanup to remove duplicate reads (MarkDuplicates) and recalibration of base quality scores (BaseRecalibrator and ApplyBQSR). Variants were called for each BAM file using HaplotypeCaller and jointly genotyped (CombineGVCFs and GenotypeGVCFs) within the family where family members were available. The raw calls were then processed to split multi-

allelic variants and re-align indels. Variants were annotated using the Ensembl Variant Effect Predictor (VEP) together with multiple external data sources (i.e., ClinVar, CADD) and underwent a series of filtering steps to remove intergenic variants, non-functional variants and common variants in the population with a Minor Allele Frequency (MAF) > 1%. Finally, the remaining calls were refined by running the inheritance-mode calling algorithms novoCaller and comHet to detect de-novo and compound heterozygous variants for probands with sequenced family members. *FGF14* non-synonymous variants were selected from the annotated VCF file generated by the pipeline. Selection criteria included variants that had a moderate or high impact with MAF < 1% in the East Asian population, or a ClinVar annotation of "pathogenic", "likely pathogenic", or "VUS". Known pathogenic repeat expansions were screened with the ExpansionHunter algorithm (v.5.0.0). A catalog of pre-defined pathogenic repeats consisting of repetitions of short sequence units (1-6 bp) was used for screening (Supplementary Table 3). The number of *FGF14* (GAA) repeats on each allele was estimated using information from reads that span, flank, and are fully contained in each repeat. Figure 1 was generated with the Python package, Matplotlib whereas Scikit-learn was used for linear regression calculations.

**Data availability**

All genome data has been shared with the MSA Coalition Collaborative Core Network (https://missionmsa.org/). This is available on request through the corresponding author, and with appropriate data use agreements and Institutional Review Board approvals. Additional anonymized genomic data is available from healthy Korean volunteers participating in the Korean Genome Project (KGPcontrols n=1,048) through the Korean National Institutes of Health.[8]

**Competing interests**

The authors report no competing interests.

## References


1. Roy S, Coldren C, Karunamurthy A, et al. Standards and Guidelines for Validating Next-Generation Sequencing Bioinformatics Pipelines: A Joint Recommendation of the Association for Molecular Pathology and the College of American Pathologists. *J Mol Diagn*. 2018;20(1):4-27. doi:10.1016/j.jmoldx.2017.11.003

2. Schneider F, Maurer C, Friedberg RC. International Organization for Standardization (ISO) 15189. *Ann Lab Med*. 2017;37(5):365-370. doi:10.3343/alm.2017.37.5.365

3. Genome in a bottle—a human DNA standard. *Nat Biotechnol*. 2015;33(7):675-675. doi:10.1038/nbt0715-675a

4. Rafehi H, Read J, Szmulewicz DJ, et al. An intronic GAA repeat expansion in FGF14 causes the autosomal-dominant adult-onset ataxia SCA50/ATX-FGF14. *Am J Hum Genet*. 2023;110(1):105-119. doi:10.1016/j.ajhg.2022.11.015

5. Lüth T, Laβ J, Schaake S, et al. Elucidating Hexanucleotide Repeat Number and Methylation within the X-Linked Dystonia-Parkinsonism (XDP)-Related SVA Retrotransposon in TAF1 with Nanopore Sequencing. *Genes (Basel)*. 2022;13(1):126. doi:10.3390/genes13010126

6. Trinh J, Luth T, Schaake S, et al. Mosaic divergent repeat interruptions in XDP influence repeat stability and disease onset. *Brain*. 2023;146(3):1075-1082. doi:10.1093/brain/awac160

7. Auwera GV der, O'Connor BD. *Genomics in the Cloud: Using Docker, GATK, and WDL in Terra*. First edition. O'Reilly; 2020.

8. Jeon S, Bhak Y, Choi Y, et al. Korean Genome Project: 1094 Korean personal genomes with clinical information. *Sci Adv*. 2020;6(22):eaaz7835. doi:10.1126/sciadv.aaz7835
